# Editor’s introduction to this issue (G&I 18:4, 2020)

**DOI:** 10.5808/GI.2020.18.4.e34

**Published:** 2020-12-29

**Authors:** Taesung Park

**Affiliations:** Department of Statistics, Seoul National University, Seoul 08826, Korea

In this issue, there are 12 articles: two Review Articles, eight Original Articles, one Research Communication, and one Application Note. The first review by Kim et al. (Dankuk University, Cheonan, Korea) is about the nature of triple-negative breast cancer (TNBC) classification and antitumoural strategy. It is well known that TNBC, characterized by estrogen receptor, progesterone receptor genes, and human epidermal growth factor receptor 2 genes, has highly aggressive clinical outcomes. The authors provided a further classification into specific subtypes according to their genomic mutation and cancer immunogenicity with its promising treatments. The second review by Seo et al. (Korea National University of Education, Cheonju, Korea) provides an overview about the roles of long non-coding RNAs (lncRNAs) and microRNAs (miRNAs) in lung cancer cells and those of the lncRNA/miRNA competing endogenous RNA networks in carcinogenesis and therapeutic resistance of lung cancer.

Among eight original articles, there are three articles related to clinical studies. The first original article by the group of Rha (Yonsei University College of Medicine, Seoul, Korea) is about gastric cancer. The authors performed transcriptome analysis of iBET-151, a BET inhibitor, which reduces the expression of oncogenes. Analysis of RNA sequencing of gastric cancer cells treated with iBET-151 and/or paclitaxel identified the differentially expressed genes associated with possible mechanisms of synergistic effect. Although additional functional studies are needed, it is the first evidence of the synergistic effect between iBET-151 and paclitaxel in regulating the transcriptome of gastric cancer cells. The second original article is about for chronotype which is an important moderator of psychiatric illnesses. Park et al. (Eulji University, Korea) investigated genetic associations and gene-gene interactions for chronotype. The clock genes such as *BHLHB2, CLOCK, CSNK1E, NR1D1, PER1, PER2, PER3*, and *TIMELESS* were successfully identified from 1,293 healthy Korean individuals via regression analysis and the quantitative multifactor dimensionality reduction method.

The third original article by Wee and Kumar (Management and Science University, Shah Alam, Malaysia) is about Alzheimer’s disease. The authors identified the hub genes of Alzheimer’s disease. Through network analysis of protein-protein interactions, the authors successfully identified the top 10 hub genes associated with Alzheimer’s disease: *PTGER3, C3AR1, NPY, ADCY2, CXCL12, CCR5, MTNR1A, CNR2, GRM2*, and *CXCL8*.

Next, Chung (The Catholic University, Seoul, Korea)’s group developed reverse transcription loop-mediated isothermal amplification (LAMP) assays for the point-of-care testing of avian influenza virus subtype H5 and H9 by adopting LAMP technology. The new sets of reverse transcription LAMP are shown to be approximately four times quicker than conventional reverse-transcription polymerase chain reaction.

The next two original articles are about an algorithm of bioinformatics and comparison studies for the bioinformatics tools. Stambler (Bar Ilan University, Ramat Gan, Israel) proposed an algorithm for the construction and analysis of autocorrelation (information) functions to identify aging and cancer-related genes based on the normalized mutual information. Roh’s group (Pohang University of Science and Technology, POSTECH, Pohang, Korea) performed a comparative study for commonly used peak calling programs for ChIP-Seq (chromatin immunoprecipitation coupled with high-throughput DNA sequencing) analysis such as five peak callers (CisGenome, MACS1, MACS2, PeakSeq, and SISSRs) using 12 publicly available ChIP-Seq data.

The coronavirus disease 2019 (COVID-19) is a highly contagious disease and created widespread mortality and morbidity. Recent efforts on COVID-19 pandemics have produced large volume of research articles. In this issue, there are three articles related to COVID-19. The first research article by Rath’s group (Odisha University of Agriculture and Technology, Bhubaneswar, India) analyzed the phylogenetic relationship of N protein sequence divergence with other 49 coronavirus species and identified the conserved regions according to protein families through conserved domain search. The authors established antiviral drug glycyrrhizic acid and the phytochemical theaflavin as possible drug compounds against target N-protein of severe acute respiratory syndrome coronavirus 2 (SARS-CoV-2) with lower binding affinities. The second article by Biswas and Mudi (Bangabandhu Sheikh Mujib Medical University, Dhaka, Bangladesh) presents the comparison results of mutation profiles of SARS-CoV-2 isolated from mildly affected and severely affected COVID-19 patients and explores relationship between mutation profile and disease severity. Through the genomic sequences of SARS-CoV-2, spike protein D614G and RNA-dependent RNA polymerase P323L mutations in SARS-CoV-2 were shown to be associated with severity of COVID-19. The final article on COVID-19 is a short research communication by Kamruzzaman et al. (Seoul National University, Seoul, Korea). It is the updated 95% confidence intervals COVID-19 antibody rate for the Korean population using three recently performed antibody tests in Korea. The most conservative 95% confidence interval estimation shows that as of 00:00 November 23, 2020, at least 69,524 people were infected but not confirmed. More positive cases were found among the young male in their twenties (0.22%), three times that of the public (0.051%).

The one article of application note by Kim’s group (Korea Research Institute of Bioscience and Biotechnology and University of Science and Technology, Daejeon,Korea) developed the BaSDAS (Barcode-Seq Data Analysis System), a GUI-based pooled knockout screening data analysis system with a user-friendly web interface. The BaSDAS facilitates the analysis of pooled knockout screen data easily and supports the analysis of various pooled screening libraries including yeast, human and mouse with many useful statistical and visualization functions.

